# Exposed Hydrophobic Residues in Human Immunodeficiency Virus Type 1 Vpr Helix-1 Are Important for Cell Cycle Arrest and Cell Death

**DOI:** 10.1371/journal.pone.0024924

**Published:** 2011-09-16

**Authors:** R. Anthony Barnitz, Benjamin Chaigne-Delalande, Diane L. Bolton, Michael J. Lenardo

**Affiliations:** 1 Laboratory of Immunology, National Institute of Allergy and Infectious Diseases, National Institutes of Health, Bethesda, Maryland, United States of America; 2 Immunology Graduate Group, University of Pennsylvania, Philadelphia, Pennsylvania, United States of America; University of Georgia, United States of America

## Abstract

The human immunodeficiency virus type 1 (HIV-1) accessory protein viral protein R (Vpr) is a major determinant for virus-induced G2/M cell cycle arrest and cytopathicity. Vpr is thought to perform these functions through the interaction with partner proteins. The NMR structure of Vpr revealed solvent exposed hydrophobic amino acids along helices 1 and 3 of Vpr, which could be putative protein binding domains. We previously showed that the hydrophobic patch along helix-3 was important for G2/M blockade and cytopathicity. Mutations of the exposed hydrophobic residues along helix-1 were found to reduce Vpr-induced cell cycle arrest and cell death as well. The levels of toxicity during virion delivery of Vpr correlated with G2/M arrest. Thus, the exposed hydrophobic amino acids in the amino-terminal helix-1 are important for the cell cycle arrest and cytopathicity functions of Vpr.

## Introduction

The HIV-1 accessory protein Vpr has many well-characterized functions and properties during HIV-1 infection that appear to be dependent on binding to partner molecules [1], [2]. Vpr is incorporated into budding HIV-1 virions [3], and within cells, Vpr mostly localizes to the nucleus [4]. These two properties are thought to allow Vpr to facilitate infection of non-dividing cells, by mediating the nuclear translocation of the viral pre-integration complex [5], [6]. This is accomplished through an interaction with a member of the nuclear transport pathway, importin-α [7]. However, the necessity for Vpr during infection of macrophages is controversial because a recent study has shown no difference in the infectivity of viruses lacking Vpr versus wild-type viruses [8]. In addition, in chemically growth-arrested cells, Vpr was found to be non-essential for infection [9]. Vpr has been reported to increase the transcriptional activity from the HIV-1 long terminal repeat (LTR) promoter, as well as several other promoters, through the binding of cellular transcription factors, such as Sp1 [10], [11], [12]. The HIV-1 mutation rate during reverse transcription is reduced by a binding of Vpr with uracil DNA glycosylase 2 and the incorporation of the latter into viral particles [13], [14]. However, besides bringing UNG2 into viral particles, Vpr is also able to reduce cellular levels of the enzyme by directly delivering it to an E3 ubiquitin ligase containing DCAF-1, DDB1, and Cul4 [15], [16], [17]. Vpr is a major effector of HIV-1 cytopathicity [18], [19], [20], [21]. Finally, Vpr is able to halt the proliferation of infected CD4^+^ T cells by causing cell cycle arrest at the G2/M phase [22], [23], [24], [25].

Vpr is a very small protein of only 14 kD (96 amino acids) and has no known enzymatic activity, no recognizable domains in the NCBI database, and no structural homologues in the Protein Data Bank (PDB), so the molecular basis of its actions is not completely understood [26]. Vpr functions are thought to depend on binding with partner proteins [1], [2]. The NMR structure of full length Vpr provides some insights into the possible mechanism of Vpr activity [27]. Vpr forms a bundle of three α-helices folded around a hydrophobic core with flexible termini. The structure reveals solvent exposed hydrophobic amino acids along helices 1 and 3 of Vpr [27]. In principle, hydrophobic patches seek to be solvent inaccessible and therefore we conjecture that a hydrophobic substance, perhaps another protein, likely interacts with these patches. Therefore, we hypothesized that these hydrophobic patches serve as protein interaction sites that might be important for Vpr functions.

Separate regions within Vpr have been shown to be critical to many Vpr functions [28]. Incorporation into budding virions has been mapped to the amino (N)-terminal half of Vpr, particularly the amphipathic helix-1 [29], [30], [31], [32], [33]. Vpr helices 1 and 2 have also been shown to be important in the nuclear localization of Vpr [29], [30], [33], [34], [35], which depends on an interaction between Vpr helix-1 and importin-α, an integral component of the nuclear import pathway [7]. However, a number of studies have highlighted a role for the carboxyl (C)-terminal portion of Vpr in nuclear localization, as well [4], [36], [37]. The ability of Vpr to increase transcription from the viral LTR promoter was mapped to the C-terminal half [38], [39]. Historically, the region containing helix-3 and the flexible C-terminus of Vpr has been shown to be necessary for G2/M cell cycle arrest [29], [30], [40]. Thus these distinct portions of Vpr appear to differentially regulate Vpr functions, likely through the binding of different partner molecules.

Recently, we published a structure-function based study exploring the role of the exposed hydrophobic residues along Vpr helix-3 in cell cycle arrest, nuclear localization, Vpr dimerization, and cell death [18]. This first look at the role of this hydrophobic patch found that these residues are indeed important for G2/M arrest. However, the role of these amino acids in cytotoxicity is less clear. The levels of death were proportional to the levels of cell cycle arrest induced by hydrophobic patch mutants during virion delivery of Vpr. However, when these same mutants were expressed during HIV-1 infection, the levels of cell death were similar to wild type Vpr, likely due to a G1 arrest of the infected cells as opposed to G2/M blockade [18].

Although the N-terminal helix-1 of Vpr has not been previously associated with cell cycle arrest, we hypothesized that the exposed hydrophobic patch along this α-helix could serve as a protein interaction region with a partner protein necessary for this function. We performed a similar structure-function based approach to test the involvement of the helix-1 exposed hydrophobic residues in cell cycle arrest and cytopathicity [18]. Mutation of these amino acids reduced G2/M arrest and cytopathicity during virion delivery of Vpr. Similar to our previous study [18], cell cycle arrest and cell death were correlated when Vpr was delivered by non-replicative virions. These data suggest that the hydrophobic patch along Vpr helix-1 is important during cell cycle arrest and cytopathicity, probably serving as a protein binding region.

## Methods

### Cell lines

HEK293T (293T) cells and Jurkat 1.9 cells [18], [41], [42] were maintained in RPMI 1640 (Lonza) that was supplemented with 10% fetal calf serum, 50 µM β-mercaptoethanol, 2.4 mM L-glutamine, and 100 U of penicillin-streptomycin/ml.

### HIV virus stock and virion delivery of Vpr

HIV-1 viral stocks were produced in 293T cells by transfection using ExGen 500 according to the manufacturer's instructions (Fermentas). Virion delivery of Vpr has been previously described [18], [41], [43], [44]. A plasmid encoding a reverse transcriptase mutant with the *vpr* gene deleted (pNL4-3_e-n-GFP_RT^m^, VprΔ22-86; a gift from E. Freed, National Cancer Institute, NIH) and a plasmid encoding the VSV-G envelope protein (pLVSV-G) were transfected into 293T cells to produce VSV-G pseudotyped virions. Plasmids encoding WT and mutant Vpr were co-transfected in order to transcomplement the Vpr-deficient virus. Jurkat cells were infected in 12-well plates in the presence of Polybrene (5 µg/ml; Sigma-Aldrich). Virus was adsorbed for 30 min at 37°C in 5% CO_2_, and then the plates were centrifuged for 30 min at 800× *g* at room temperature.

### Transfection

An Electro-Cell manipulator (BTX) apparatus was used to transiently transfect Jurkat cells by electroporation. A total of 4×10^6^ cells were resuspended to a concentration of 10×10^6^ cells/ml with 10 to 15 µg of DNA in a 4-mm gap cuvette (Bio-Rad Laboratories) and electroporated at 260 V and 1,060 µF. Transfected cells were transferred to fresh supplemented RPMI and assayed after 3 days. A plasmid encoding the green fluorescent protein (GFP) was cotransfected at a 1∶5 ratio to the Vpr expression plasmid to identify transfected cells. The human codon-optimized Vpr plasmid, hVpr [18], [41], was used to express WT Vpr. Mutations of W18 and L22 were generated using a PfuUltra II (Stratagene) site-directed mutagenesis protocol.

### Assays for cell cycle and cell viability

DNA content analysis was performed by propidium iodide (PI) staining. Cells were fixed with 1% paraformaldehyde in phosphate-buffered saline (PBS) for 10 min at room temperature, washed in PBS, and incubated in 70% ethanol for at least 30 min. Cells were washed again in PBS and stained with DNA staining solution (5 µg of PI/ml, 50 µg of RNase/ml, and 0.45 mg of sodium citrate/ml in PBS) at room temperature for 30 min. Stained cells were examined using a FACSCalibur flow cytometer (Becton Dickinson), and a constant number of cells were measured. Viability of infected cells was assessed by the exclusion of the vital dye propidium iodide, measured for a constant period of time (30 sec) per sample. All flow cytometric data were analyzed by using FlowJo software (Tree Star, Inc.).

### Immunoblotting

Jurkat cells, 293T cells, and concentrated virus stocks were lysed in a 2% sodium dodecyl sulfate (SDS) buffer (2% SDS and 10% glycerol in 60 mM Tris-HCl [pH 7.5] with 1 U/µl of DNase [Benzonase nuclease; Novagen] and Complete protease inhibitor cocktail [Roche]) for at least 30 min at 4°C. A bicinchoninic acid assay (Pierce) was used to determine protein concentration of the lysates. Equal masses of protein were loaded onto a 4 to 20% Bis-Tris SDS gel (Bio-Rad Laboratories) for SDS-polyacrylamide gel electrophoresis, and protein was transferred to nitrocellulose using a semidry transfer apparatus (Bio-Rad Laboratories). Nitrocellulose blots were blocked in 5% nonfat milk in 0.1% PBS-Tween 20 (PBS-T). Blots were probed with primary antibody overnight at 4°C, followed by secondary antibody conjugated to horseradish peroxidase diluted 1∶5,000. All antibodies were diluted in 5% nonfat milk in PBS-T. After each antibody incubation, blots were washed three times in PBS-T. The bands were imaged by using enhanced chemiluminescent or SuperSignal West Dura substrates (Pierce). Densitometry was performed using ImageJ software (NIH). The primary antibodies used include Vpr antiserum (a gift from K. Strebel), p24-capsid (AIDS Research and Reference Reagent Program; 6457), and β-actin (Sigma-Aldrich; A1978).

## Results

**Figure 1:**
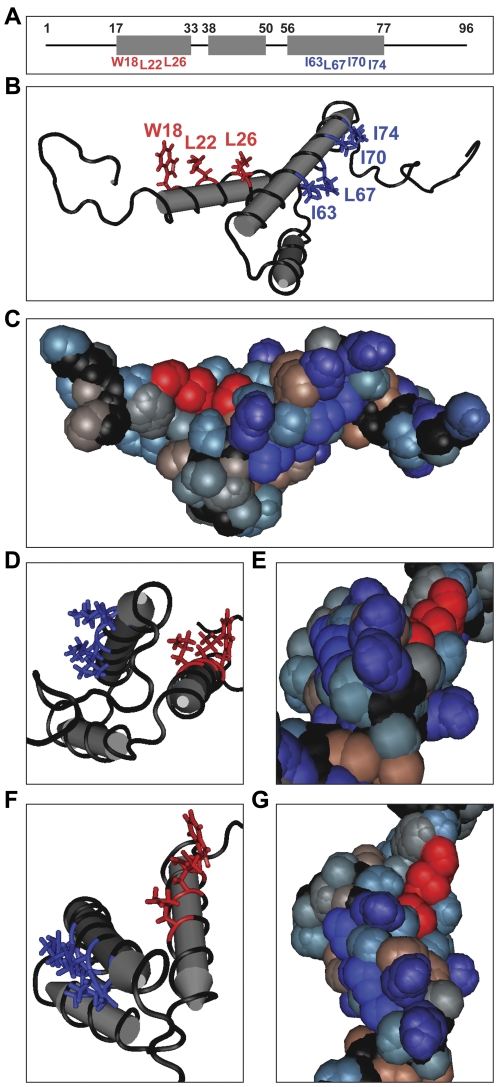
The NMR structure of Vpr shows solvent exposed hydrophobic residues along helices 1 and 3. (**A**) A wire and box diagram of Vpr. The gray boxes represent the 3 helices of Vpr, and the upper set of numbers denotes the boundaries of each helix. The lower set of numbers identifies exposed hydrophobic residues along helix 1 and helix 3. (**B**) A tube diagram of the side view of the nuclear magnetic resonance (NMR) structure of HIV-1 Vpr (PDB: 1M8L) indicating hydrophobic amino acids exposed on the apparently solvent exposed surfaces of helix 1 (red) and helix 3 (blue). (**C**) A space filling diagram of the side view of HIV-1 Vpr using a hydrophobicity color scheme. The exposed hydrophobic residues along helices 1 and 3 are colored as in (B). Positively charged or polar amino acids with high hydrophilicity are colored light blue. Purple residues are negatively charged with high hydrophilicity. Polar amino acids with low to neutral hydrophobicity are gray, and highly hydrophobic, nonpolar residues are brown. The α-carbon peptide chain is black as in (B). (**D**) C-terminal end view of HIV-1 Vpr helical core indicating the hydrophobic patches colored as in (B). (**E**) A space filling end view as in (D) colored as in (C). (**F**) Top view of HIV-1 Vpr helical core indicating the hydrophobic patches colored as in (B). (**G**) Top view of HIV-1 Vpr using a space filling diagram colored as in (C).

The core of the Vpr protein (amino acids [AA] 17–77) is comprised of three α-helices folded around a hydrophobic core [27]. Figure 1A shows the boundaries of Vpr helix-1 (AA 17–33), helix-2 (AA 38–50), and helix-3 (AA 56–77) [27]. The animo (N)- and carboxyl (C)-termini were found to be flexible and unstructured (Figure 1). Along helices 1 and 3 of Vpr are several solvent-exposed hydrophobic amino acids (Figure 1, red for amino acids of helix-1 and blue for those of helix-3). The rest of the Vpr surface is hydrophilic, with only a few sporadically spaced hydrophobic amino acids partially exposed to the solvent environment (Figure 1C, E, and G). Using a previously successful strategy [18], we employed a structurally based mutagenesis approach to disrupt the hydrophobic patch on Vpr helix-1. The residues tryptophan (W) 18 and leucine (L) 22 were mutated to alanine (A) (small, non-polar), serine (S) (polar), or glutamic acid (E) (charged, highly hydrophilic) in order to disrupt the chemical nature of the hydrophobic patch to varying degrees. Although L26 is also exposed to the surface of the protein, the side chain has lipophilic interactions with the core of the protein as well [27] (Figure 1C, E, and G). Therefore, mutations of L26 would probably destabilize the entire protein. We attempted to minimize any possible disruption to the secondary and tertiary structure of Vpr by only mutating amino acids with outward facing side groups.

**Figure 2:**
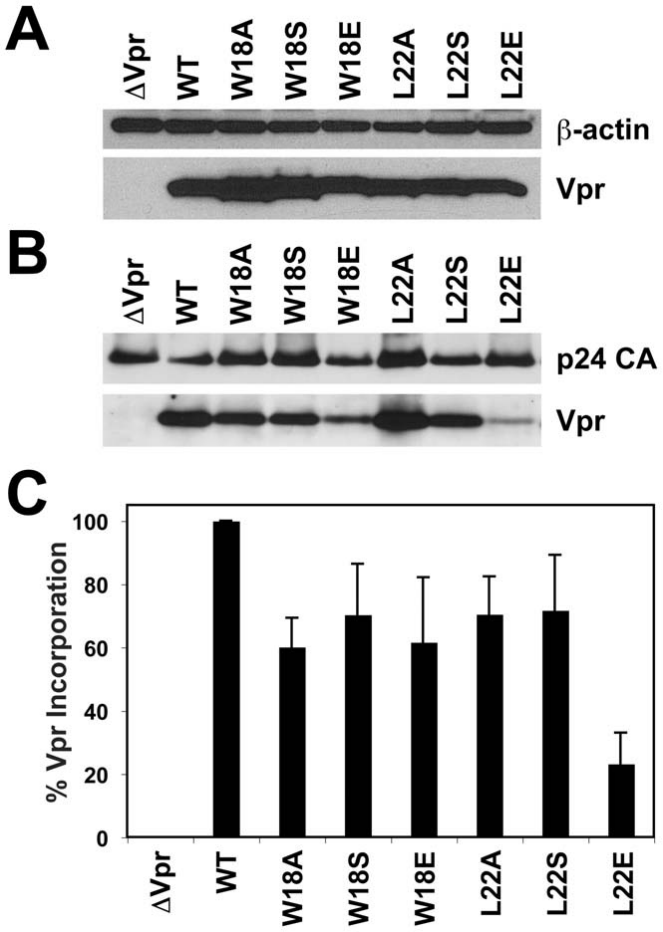
Hydrophobic residues on Vpr helix-1 are important for incorporation into virions. (**A**) 293T cells were co-transfected with pcDNA3-hVpr plasmids expressing WT or mutant Vpr (or the empty vector control), pLVSV-G, and pNL4-3_e-n-GFP RTm, VprΔ22-86_ to produce virus for virion delivery of Vpr. At day 2 the virus stocks were harvested and the 293T cells were lysed. Western blot indicates protein levels for WT or mutant Vpr (bottom). A western blot for β-actin is used as a loading control (top). (**B**) Lysates were prepared from the virus stocks in (A). Viral lysates were analyzed for protein levels of WT or mutant Vpr by western blotting as in (A) (bottom). The HIV-1 p24 capsid (p24 CA) is shown as a protein loading control (top). (**C**) Densitometry of all bands in (B) was performed. The intensity of the Vpr protein band was normalized to p24 CA and plotted as % Vpr incorporation relative to WT Vpr for each mutant. The data are shown as the mean ± the standard deviation of three independent experiments.

In the previous analysis, some of the Vpr helix-3 mutants could not be expressed [18]. Therefore, we first tested the expression of the helix-1 mutants by western blot analysis as previously described [41]. All Vpr mutants expressed at similar levels as WT Vpr in HEK293T cells (Figure 2A). Since helix-1 of Vpr is responsible for the incorporation into budding virions by binding to the p6 region of Gag [30], [31], [32], [45], we also studied the helix-1 mutants for Vpr incorporation by producing non-replicative virus using a reverse transcriptase mutant and Vpr deficient strain of HIV-1 (NL4-3_e-n-GFP_RT^m^, VprΔ22-86) and trans-complementing Vpr with separate expression plasmids as previously described [18], [41]. Although expression was comparable in the 293T producer cells, WT and mutant Vpr were differentially packaged into virions (Figure 2B–C). We calculated the percent of Vpr that is incorporated into the virions (relative to WT Vpr) by comparing the relative intensities (by densitometry analysis of the western blots) of the Vpr protein bands to the p24 capsid structural protein. All of the Vpr helix-1 mutants were packaged into virions at approximately 40–80% of the level of WT, except for L22E, which was only ∼20% compared to WT (Figure 2C).

**Figure 3:**
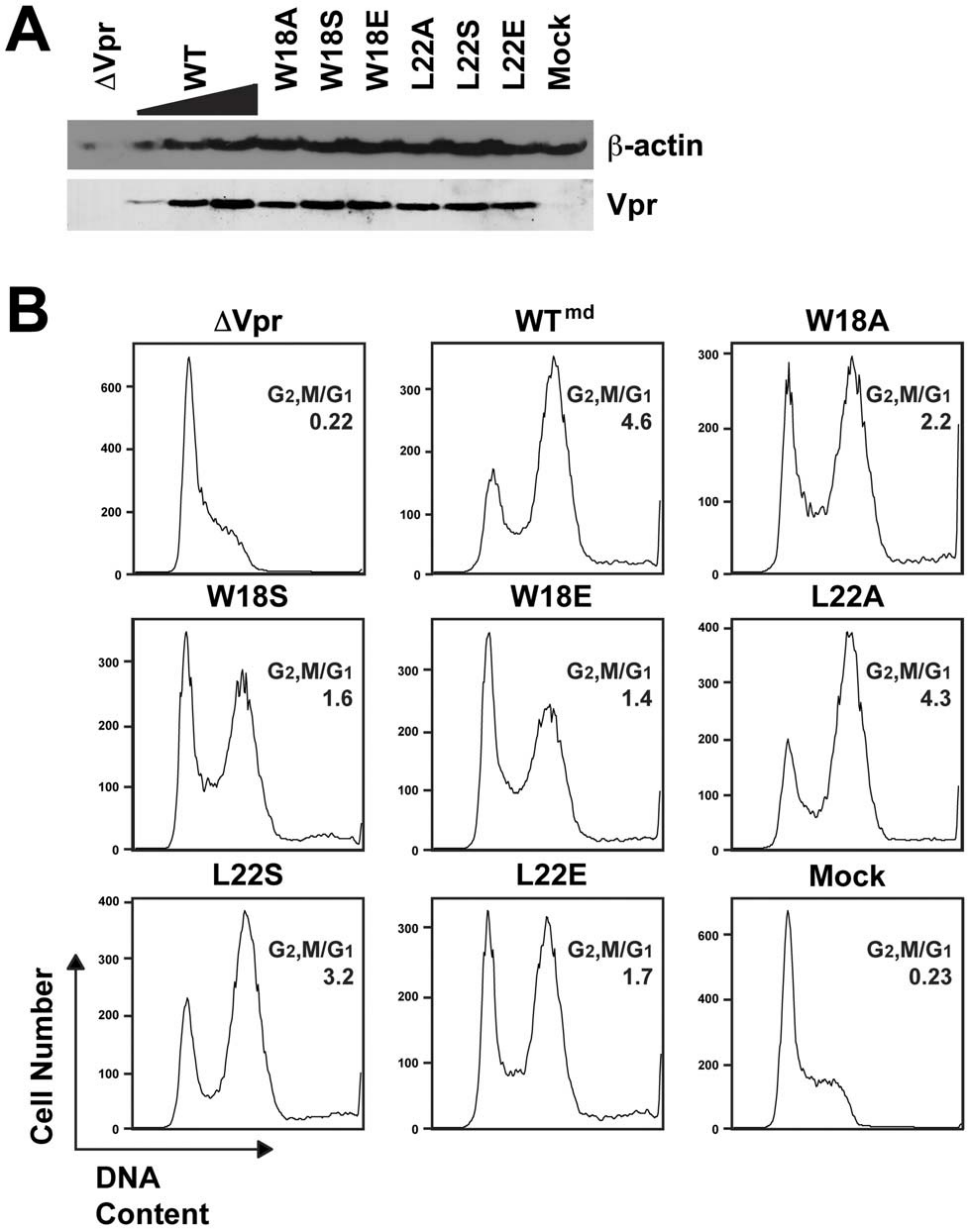
Hydrophobic residues on Vpr helix-1 are important for virion-delivered Vpr cell cycle arrest. WT or mutant Vpr proteins (denoted by the single letter amino acid changes) were delivered (Vpr_v_) into Jurkat cells. Virions containing WT Vpr were titrated (md, medium) so that a matched Vpr protein control could be compared to the mutants. (**A**) Western blot of the Jurkat cells for WT and mutant virion-delivered Vpr (bottom). β-actin is shown as a protein loading control (top). (**B**) Histograms of cell cycle analysis at 41 hr post-infection show DNA content of PI-stained cells by flow cytometry. All samples represent 10,000 cellular events. G1 and G2/M populations were modeled using the Watson Pragmatic cell cycle model, and the G2,M/G1 ratio in each infection is shown. The data are representative of three experiments.

We first characterized the cell cycle arrest activity of the helix-1 mutants by using virion delivery of Vpr (Vpr_v_) as previously described [18], [41]. We titrated the amount of WT Vpr_v_ to provide matched control levels of Vpr for the mutants. All of the mutants were delivered into the Jurkat cells at similar levels as the medium dose of WT Vpr_v_ (G2,M/G1 = 4.6) by western blot analysis (Figure 3A). Interestingly, mutation of W18 to any of the substitute residues (A, S, or E) greatly reduced the Vpr-induced G2/M arrest (Figure 3B). Glutamic acid had the greatest effect (G2,M/G1 = 1.4), as predicted, since the negatively charged glutamic acid should disrupt the chemical nature of the hydrophobic patch to the greatest degree. W18 may assist the binding to a partner protein through stacking interactions of the aromatic ring structure, which could explain why all mutations of this residue had a pronounced effect. The alanine substitution of L22 had no effect on Vpr_v_ cell cycle arrest. However, the L22S (G2,M/G1 = 3.2) and the L22E (G2,M/G1 = 1.7) mutations reduced the G2/M arrest (Figure 3A).

**Figure 4:**
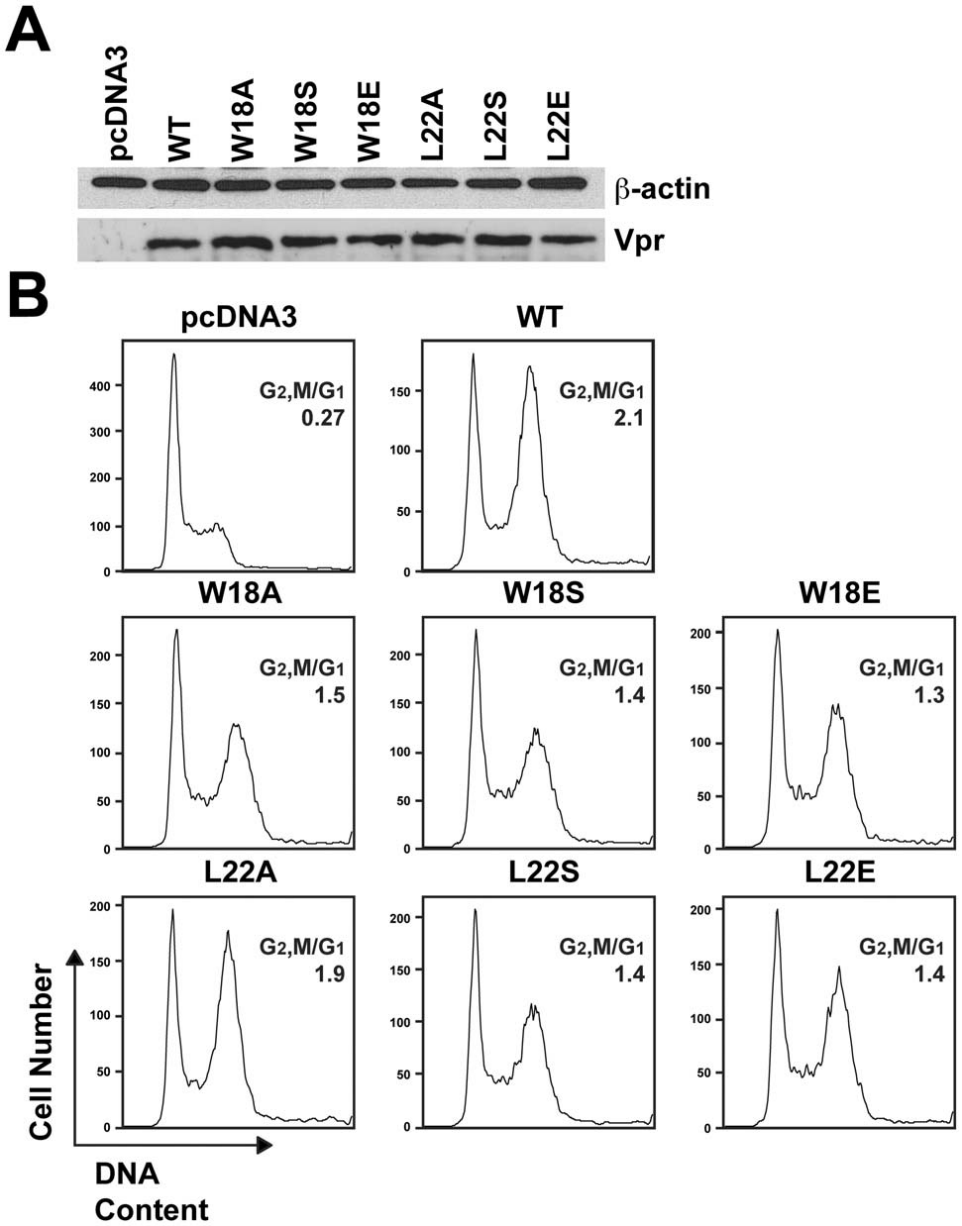
Exposed hydrophobic residues on Vpr helix-1 are important for transfected Vpr G2/M arrest. Jurkat cells were co-transfected with pcDNA3-hVpr plasmids expressing WT or mutant Vpr (or the empty vector control) and pEGFP-N1 as a transfection marker at a ratio of 5∶1. (**A**) Western blot of the Jurkat cells for WT or mutant Vpr (bottom). A western blot of β-actin was used as a protein loading control (top). (**B**) Histograms of cell cycle analysis at 61 hr post-transfection show DNA content of GFP+, PI-stained cells by flow cytometry. All samples represent 10,000 cellular events. G1 and G2/M populations were modeled using the Watson Pragmatic cell cycle model, and the G2,M/G1 ratio in each transfection is shown. The data are representative of three experiments.

Transient transfection can also be used to evaluate Vpr-induced cell cycle arrest [18], [41]. Equivalent expression of the mutants was confirmed by western blot analysis, and all of the mutants were expressed at a similar level as the WT sample relative to the loading control (Figure 4A). The same cell cycle trend was found with transfection as with virion delivery. All substitutions for W18 reduced cell cycle arrest. Whereas the L22A mutation had minimal effect, mutations to S and E both showed less accumulation of cells in G2/M than WT (Figure 4B). These results indicate that the exposed hydrophobic patch on Vpr helix-1 is important for cell cycle arrest function, corroborating the virion-delivered Vpr_v_ system.

**Figure 5:**
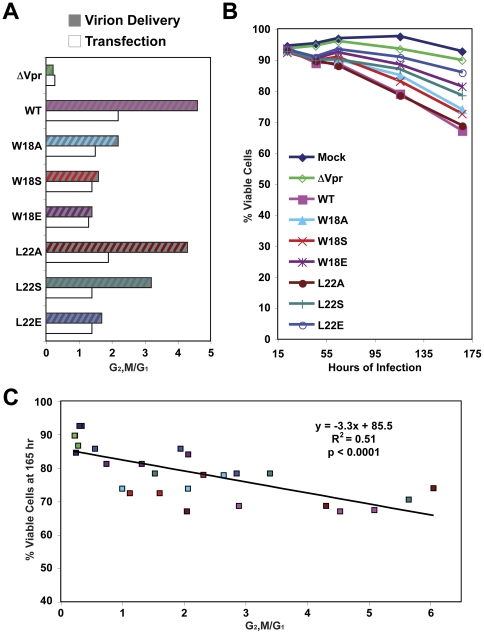
Vpr G2/M arrest correlates with cell death induced by virion delivery. (**A**) WT or mutant Vpr proteins (denoted by the single letter amino acid changes) were delivered (Vpr_v_) into Jurkat cells, and separately Jurkat cells were co-transfected with pcDNA3-hVpr plasmids expressing WT or mutant Vpr (or the empty vector control) and pEGFP-N1 as a transfection marker at a ratio of 5∶1. Infected and transfected cells were analyzed for DNA content of the PI-stained cells by flow cytometry. G1 and G2/M populations were modeled using the Watson Pragmatic cell cycle model, and the G2,M/G1 ratios were plotted on the x-axis. (**B**) The viability of the Vpr_v_-treated cells was monitored over time by flow cytometry (detection of PI-negative, forward-scatter high events), and the percentage of viable cells is plotted over time. These data are representative of three experiments. (**C**) The G2,M/G1 ratios and viability of Vpr_v_-treated cells at 165 hour post-infection from three independent experiments were plotted. Each measurement is color-coded as in (B). Spearman's rank test was used to determine the correlation.

The previous study on the helix-3 hydrophobic patch (I63, I67, and I74) found a correlation between cell cycle arrest and cytopathicity for the mutants using virion delivered Vpr_v_ [18]. We examined the helix-1 mutants for a similar correlation. WT and mutant Vpr was delivered into Jurkat cells, and the cell cycle profile and viability of these cells were assessed over time as previously described (Figure 5) [18]. Similar to the previous report, we found that wild type Vpr_v_ reduced the cell viability to approximately 65% over the course of infection (Figure 5B). G2/M arrest correlated with the toxicity of the helix-1 mutants during virion delivery (Figure 5C). The L22A mutant, which has comparable cell cycle arrest function as WT, also showed similar cell death activity (Figure 5). The other mutants exhibited reduced cytopathicity proportional to the reduction in cell cycle arrest. The W18E and L22E mutants showed the most pronounced reduction in toxicity, corresponding with the most attenuation of G2/M arrest (Figure 5). Although cell cycle arrest correlated with cell death among the Vpr mutants, some discrepancies still existed. For example, Vpr L22S caused more arrest than both W18A and W18S (Figure 5A); yet L22S caused less death than W18A/S (Figure 5B). These results suggest that the exposed hydrophobic residues along Vpr helix-1 are important for cytopathicity, and that G2/M arrest frequently correlates with cell death, similar to the results with the helix-3 mutants [18].

## Discussion

Vpr likely functions by binding with partner proteins [1], [2], [28], and our lab has previously shown a role for the exposed hydrophobic patch along Vpr helix-3, an expected protein-protein interaction site, in the G2/M cell cycle arrest and cell death functions [18]. Here, we present an analysis of another expected protein binding domain, the hydrophobic patch of Vpr helix-1, for Vpr-induced G2/M arrest and cytopathicity, as well as virion incorporation. Although, the expected defect in virion incorporation was observed with the mutations we created, this is the first examination specifically of the hydrophobic patch residues. The L22E mutant was particularly impaired in virion incorporation, suggesting that the introduction of a negative charge at this site inhibits the binding of Vpr to the p6 region of the Gag polyprotein. We found that the exposed hydrophobic amino acids W18 and L22 are important for both G2/M arrest and cytopathicity, and that these two properties of Vpr are correlated.

We found that specific mutants of W18 and L22 reduced cell cycle arrest to varying degrees in both virion delivery of Vpr and transient transfection. The differences between the mutations to A, S, and E at each position were most apparent with Vpr_v_ due to the higher degree of cell cycle arrest caused by this technique. However, even though the differences were smaller using transfection, the same trend was seen between the mutants (Figures 4 and 5A). The correlation between G2/M arrest and Vpr-induced death caused by Vpr_v_ is similar to our previous study of Vpr helix-3 [18]. However, analysis of the helix-3 mutants in the context of HIV-1 infection did not show reduced cytopathicity. This was due to a G1 arrest caused by the mutant Vpr. Even though the infected cells exhibited a more normal DNA content profile, the cells were still inhibited from proliferating [18]. It would be interesting to test whether the helix-1 mutants relieve G2/M arrest but promote a G1 arrest during HIV-1 infection, and thus still kill the infected cell.

Vpr is thought to mediate all of its known functions through the binding of partner molecules [26], [28]. We and others have shown that Vpr binds to the 14-3-3 family of scaffold proteins [42], [46]. This interaction induces a large molecular complex nucleated on the 14-3-3 protein that consists of a number of cell cycle regulatory proteins [42]. Recently, multiple groups have found that Vpr binds to an E3 ubiquitin ligase comprised of DCAF1/VprBP, DDB1, Cul4, and Roc1 [16], [47], [48], [49], [50], [51], [52]. Vpr is proposed to recruit an unknown but critical cell cycle component into the E3 ligase complex and induce the abnormal degradation of that unknown cell cycle protein. This mechanism has already been shown to occur for the UNG2 protein [15], [16], [17]. Perhaps the hydrophobic patch of Vpr helix-1 and/or helix-3 participates in the interaction between Vpr and any of these partner proteins. Thus, it would be interesting to examine the role of exposed hydrophobic residues along these α-helices (W18, L22, I63, L67, and I74) in Vpr-dependent molecular complexes.

A close examination of our data and the results of the helix-3 study reveals that none of the mutants completely abrogated G2/M arrest [18]. However, all of these mutants only substitute a single residue. It is possible that a single change in either of the hydrophobic patches is not enough of a disruption of the chemical nature of the putative protein interaction site. Therefore, testing combinations of mutants, such as W18A and L22A, may further reduce cell cycle arrest and cell death cause by Vpr. Another possibility is that the exposed hydrophobic amino acids along both Vpr helices could actually comprise one protein interaction site. The hydrophobic side groups do orient to the same outward “half” of the core of Vpr (Figure 1). However, the “ridge” of arginines along helix-3 (Figure 1C, E, and G) would likely disrupt the continuity of a single large hydrophobic patch. Perhaps mutations in both patches are necessary to completely inhibit the G2/M arrest function.

We have previously shown that HIV-1 causes a necrotic death of the host CD4^+^ T cells and T cell lines [53], [54]. The accessory proteins Vpr and Vif are independently able to cause this cytotoxicity [19]. Interestingly, both Vpr and Vif are able to induce a G2/M cell cycle arrest, suggesting that cell cycle arrest may be the cause of HIV-induced necrosis [19], [42]. In fact, mutants of the exposed hydrophobic residues of Vpr helix-3 cause a G1 cell cycle arrest in the context of HIV-1 infection. This G1 arrest still leads to the death of the host cell, indicating that any inhibition of proliferation of HIV-1 infected cells will cause the death of the cell [18]. It would be interesting to test whether the helix-1 mutants also induced a G1 arrest in the context HIV-1 infection leading to cell death. Further investigation into the exact mechanism linking cell cycle arrest to cytopathicity could provide possible targets for therapies that would reduce the depletion of CD4^+^ T cells during HIV-1 infection.

We found that the exposed hydrophobic amino acids W18 and L22 are important for both G2/M arrest and cytopathicity, and that these two properties of Vpr are correlated. Although this is not the first study to implicate the amino-terminal region of Vpr in cell cycle arrest, there are unresolved questions in the previous mutagenesis findings. Mutations of hydrophobic core residues, such as A30L [29] and H33R [40], would likely cause misfolding of the protein around the core [27]. Introduction of proline residues into the first α-helix, such as the E21,24P mutation [30], would destroy the helical structure of that region, and probably alter the entire global structure of Vpr [26]. We cannot be certain that the W18 and L22 mutations that we introduced did not significantly alter Vpr structure without further structural examination. However, these residues do not interact with the hydrophobic core of the protein, and disruption of the local secondary structure by the substitute residues is unlikely. Therefore, this is the first study showing a role for the helix-1 hydrophobic patch in Vpr-induced G2/M arrest and cytotoxicity.

## References

[1] Kino T, Pavlakis GN (2004). Partner molecules of accessory protein Vpr of the human immunodeficiency virus type 1.. DNA Cell Biol.

[2] Le Rouzic E, Benichou S (2005). The Vpr protein from HIV-1: distinct roles along the viral life cycle.. Retrovirology.

[3] Cohen EA, Dehni G, Sodroski JG, Haseltine WA (1990). Human immunodeficiency virus vpr product is a virion-associated regulatory protein.. J Virol.

[4] Lu YL, Spearman P, Ratner L (1993). Human immunodeficiency virus type 1 viral protein R localization in infected cells and virions.. J Virol.

[5] Connor RI, Chen BK, Choe S, Landau NR (1995). Vpr is required for efficient replication of human immunodeficiency virus type-1 in mononuclear phagocytes.. Virology.

[6] Heinzinger NK, Bukinsky MI, Haggerty SA, Ragland AM, Kewalramani V (1994). The Vpr protein of human immunodeficiency virus type 1 influences nuclear localization of viral nucleic acids in nondividing host cells.. Proc Natl Acad Sci U S A.

[7] Kamata M, Nitahara-Kasahara Y, Miyamoto Y, Yoneda Y, Aida Y (2005). Importin-alpha promotes passage through the nuclear pore complex of human immunodeficiency virus type 1 Vpr.. J Virol.

[8] Riviere L, Darlix JL, Cimarelli A (2010). Analysis of the viral elements required in the nuclear import of HIV-1 DNA.. J Virol.

[9] Yamashita M, Emerman M (2005). The cell cycle independence of HIV infections is not determined by known karyophilic viral elements.. PLoS Pathog.

[10] Cohen EA, Terwilliger EF, Jalinoos Y, Proulx J, Sodroski JG (1990). Identification of HIV-1 vpr product and function.. J Acquir Immune Defic Syndr.

[11] Felzien LK, Woffendin C, Hottiger MO, Subbramanian RA, Cohen EA (1998). HIV transcriptional activation by the accessory protein, VPR, is mediated by the p300 co-activator.. Proc Natl Acad Sci U S A.

[12] Wang L, Mukherjee S, Jia F, Narayan O, Zhao LJ (1995). Interaction of virion protein Vpr of human immunodeficiency virus type 1 with cellular transcription factor Sp1 and trans-activation of viral long terminal repeat.. J Biol Chem.

[13] Chen R, Le Rouzic E, Kearney JA, Mansky LM, Benichou S (2004). Vpr-mediated incorporation of UNG2 into HIV-1 particles is required to modulate the virus mutation rate and for replication in macrophages.. J Biol Chem.

[14] Mansky LM, Preveral S, Selig L, Benarous R, Benichou S (2000). The interaction of vpr with uracil DNA glycosylase modulates the human immunodeficiency virus type 1 In vivo mutation rate.. J Virol.

[15] Ahn J, Vu T, Novince Z, Guerrero-Santoro J, Rapic-Otrin V (2010). HIV-1 Vpr loads uracil DNA glycosylase-2 onto DCAF1, a substrate recognition subunit of a cullin 4A-ring E3 ubiquitin ligase for proteasome-dependent degradation.. J Biol Chem.

[16] Schröfelbauer B, Hakata Y, Landau NR (2007). HIV-1 Vpr function is mediated by interaction with the damage-specific DNA-binding protein DDB1.. Proc Natl Acad Sci U S A.

[17] Schrofelbauer B, Yu Q, Zeitlin SG, Landau NR (2005). Human immunodeficiency virus type 1 Vpr induces the degradation of the UNG and SMUG uracil-DNA glycosylases.. J Virol.

[18] Bolton DL, Lenardo MJ (2007). Vpr cytopathicity independent of G2/M cell cycle arrest in human immunodeficiency virus type 1-infected CD4+ T cells.. J Virol.

[19] Sakai K, Dimas J, Lenardo MJ (2006). The Vif and Vpr accessory proteins independently cause HIV-1-induced T cell cytopathicity and cell cycle arrest.. Proc Natl Acad Sci U S A.

[20] Stewart SA, Poon B, Jowett JB, Chen IS (1997). Human immunodeficiency virus type 1 Vpr induces apoptosis following cell cycle arrest.. J Virol.

[21] Yao XJ, Mouland AJ, Subbramanian RA, Forget J, Rougeau N (1998). Vpr stimulates viral expression and induces cell killing in human immunodeficiency virus type 1-infected dividing Jurkat T cells.. J Virol.

[22] He J, Choe S, Walker R, Di Marzio P, Morgan DO (1995). Human immunodeficiency virus type 1 viral protein R (Vpr) arrests cells in the G2 phase of the cell cycle by inhibiting p34cdc2 activity.. J Virol.

[23] Jowett JB, Planelles V, Poon B, Shah NP, Chen ML (1995). The human immunodeficiency virus type 1 vpr gene arrests infected T cells in the G2+M phase of the cell cycle.. J Virol.

[24] Re F, Braaten D, Franke EK, Luban J (1995). Human immunodeficiency virus type 1 Vpr arrests the cell cycle in G2 by inhibiting the activation of p34cdc2-cyclin B.. J Virol.

[25] Rogel ME, Wu LI, Emerman M (1995). The human immunodeficiency virus type 1 vpr gene prevents cell proliferation during chronic infection.. J Virol.

[26] Pandey RC, Datta D, Mukerjee R, Srinivasan A, Mahalingam S (2009). HIV-1 Vpr: a closer look at the multifunctional protein from the structural perspective.. Curr HIV Res.

[27] Morellet N, Bouaziz S, Petitjean P, Roques BP (2003). NMR structure of the HIV-1 regulatory protein VPR.. J Mol Biol.

[28] Morellet N, Roques BP, Bouaziz S (2009). Structure-function relationship of Vpr: biological implications.. Curr HIV Res.

[29] Di Marzio P, Choe S, Ebright M, Knoblauch R, Landau NR (1995). Mutational analysis of cell cycle arrest, nuclear localization and virion packaging of human immunodeficiency virus type 1 Vpr.. J Virol.

[30] Mahalingam S, Ayyavoo V, Patel M, Kieber-Emmons T, Weiner DB (1997). Nuclear import, virion incorporation, and cell cycle arrest/differentiation are mediated by distinct functional domains of human immunodeficiency virus type 1 Vpr.. J Virol.

[31] Mahalingam S, Khan SA, Jabbar MA, Monken CE, Collman RG (1995). Identification of residues in the N-terminal acidic domain of HIV-1 Vpr essential for virion incorporation.. Virology.

[32] Mahalingam S, Khan SA, Murali R, Jabbar MA, Monken CE (1995). Mutagenesis of the putative alpha-helical domain of the Vpr protein of human immunodeficiency virus type 1: effect on stability and virion incorporation.. Proc Natl Acad Sci U S A.

[33] Yao XJ, Subbramanian RA, Rougeau N, Boisvert F, Bergeron D (1995). Mutagenic analysis of human immunodeficiency virus type 1 Vpr: role of a predicted N-terminal alpha-helical structure in Vpr nuclear localization and virion incorporation.. J Virol.

[34] Kamata M, Aida Y (2000). Two putative alpha-helical domains of human immunodeficiency virus type 1 Vpr mediate nuclear localization by at least two mechanisms.. J Virol.

[35] Nie Z, Bergeron D, Subbramanian RA, Yao XJ, Checroune F (1998). The putative alpha helix 2 of human immunodeficiency virus type 1 Vpr contains a determinant which is responsible for the nuclear translocation of proviral DNA in growth-arrested cells.. J Virol.

[36] Sherman MP, de Noronha CM, Heusch MI, Greene S, Greene WC (2001). Nucleocytoplasmic shuttling by human immunodeficiency virus type 1 Vpr.. J Virol.

[37] Zhou Y, Lu Y, Ratner L (1998). Arginine residues in the C-terminus of HIV-1 Vpr are important for nuclear localization and cell cycle arrest.. Virology.

[38] Sawaya BE, Khalili K, Gordon J, Srinivasan A, Richardson M (2000). Transdominant activity of human immunodeficiency virus type 1 Vpr with a mutation at residue R73.. J Virol.

[39] Thotala D, Schafer EA, Tungaturthi PK, Majumder B, Janket ML (2004). Structure-functional analysis of human immunodeficiency virus type 1 (HIV-1) Vpr: role of leucine residues on Vpr-mediated transactivation and virus replication.. Virology.

[40] Chen M, Elder RT, Yu M, O'Gorman MG, Selig L (1999). Mutational analysis of Vpr-induced G2 arrest, nuclear localization, and cell death in fission yeast.. J Virol.

[41] Barnitz RA, Wan F, Tripuraneni V, Bolton DL, Lenardo MJ (2010). Protein kinase A phosphorylation activates Vpr-induced cell cycle arrest during human immunodeficiency virus type 1 infection.. J Virol.

[42] Bolton DL, Barnitz RA, Sakai K, Lenardo MJ (2008). 14-3-3 theta binding to cell cycle regulatory factors is enhanced by HIV-1 Vpr.. Biol Direct.

[43] Poon B, Grovit-Ferbas K, Stewart SA, Chen IS (1998). Cell cycle arrest by Vpr in HIV-1 virions and insensitivity to antiretroviral agents.. Science.

[44] Stewart SA, Poon B, Jowett JB, Xie Y, Chen IS (1999). Lentiviral delivery of HIV-1 Vpr protein induces apoptosis in transformed cells.. Proc Natl Acad Sci U S A.

[45] Bachand F, Yao XJ, Hrimech M, Rougeau N, Cohen EA (1999). Incorporation of Vpr into human immunodeficiency virus type 1 requires a direct interaction with the p6 domain of the p55 gag precursor.. J Biol Chem.

[46] Kino T, Gragerov A, Valentin A, Tsopanomihalou M, Ilyina-Gragerova G (2005). Vpr protein of human immunodeficiency virus type 1 binds to 14-3-3 proteins and facilitates complex formation with Cdc25C: implications for cell cycle arrest.. J Virol.

[47] Belzile JP, Duisit G, Rougeau N, Mercier J, Finzi A (2007). HIV-1 Vpr-mediated G2 arrest involves the DDB1-CUL4AVPRBP E3 ubiquitin ligase.. PLoS Pathog.

[48] DeHart JL, Zimmerman ES, Ardon O, Monteiro-Filho CM, Arganaraz ER (2007). HIV-1 Vpr activates the G2 checkpoint through manipulation of the ubiquitin proteasome system.. Virol J.

[49] Hrecka K, Gierszewska M, Srivastava S, Kozaczkiewicz L, Swanson SK (2007). Lentiviral Vpr usurps Cul4-DDB1[VprBP] E3 ubiquitin ligase to modulate cell cycle.. Proc Natl Acad Sci U S A.

[50] Le Rouzic E, Belaidouni N, Estrabaud E, Morel M, Rain JC (2007). HIV1 Vpr arrests the cell cycle by recruiting DCAF1/VprBP, a receptor of the Cul4-DDB1 ubiquitin ligase.. Cell Cycle.

[51] Tan L, Ehrlich E, Yu XF (2007). DDB1 and Cul4A are required for human immunodeficiency virus type 1 Vpr-induced G2 arrest.. J Virol.

[52] Wen X, Duus KM, Friedrich TD, de Noronha CM (2007). The HIV1 protein Vpr acts to promote G2 cell cycle arrest by engaging a DDB1 and Cullin4A-containing ubiquitin ligase complex using VprBP/DCAF1 as an adaptor.. J Biol Chem.

[53] Bolton DL, Hahn BI, Park EA, Lehnhoff LL, Hornung F (2002). Death of CD4(+) T-cell lines caused by human immunodeficiency virus type 1 does not depend on caspases or apoptosis.. J Virol.

[54] Lenardo MJ, Angleman SB, Bounkeua V, Dimas J, Duvall MG (2002). Cytopathic killing of peripheral blood CD4(+) T lymphocytes by human immunodeficiency virus type 1 appears necrotic rather than apoptotic and does not require env.. J Virol.

[55] Fouchier RA, Meyer BE, Simon JH, Fischer U, Malim MH (1997). HIV-1 infection of non-dividing cells: evidence that the amino-terminal basic region of the viral matrix protein is important for Gag processing but not for post-entry nuclear import.. EMBO J.

[56] Simon JH, Fouchier RA, Southerling TE, Guerra CB, Grant CK (1997). The Vif and Gag proteins of human immunodeficiency virus type 1 colocalize in infected human T cells.. J Virol.

